# The Circulating miR-107 as a Potential Biomarker Up-Regulated in Castration-Resistant Prostate Cancer

**DOI:** 10.3390/ncrna10050047

**Published:** 2024-08-24

**Authors:** Jonathan Puente-Rivera, David Alejandro De la Rosa Pérez, Stephanie I. Nuñez Olvera, Elisa Elvira Figueroa-Angulo, José Gadú Campos Saucedo, Omar Hernández-León, María Elizbeth Alvarez-Sánchez

**Affiliations:** 1 Posgrado en Ciencias Genómicas, Universidad Autónoma de la Ciudad de México (UACM), San Lorenzo 290, Col. Del Valle, México City 03100, Mexico; jo_puenter@hotmail.com (J.P.-R.); delarosaperezdavidalejandro@gmail.com (D.A.D.l.R.P.); figueroaelisaelvira@gmail.com (E.E.F.-A.); 2 División de Investigación, Hospital Juárez de México, Instituto Politécnico Nacional 5160, Col. Magdalena de las Salinas, México City 07360, Mexico; 3 Departamento de Biología Celular y Fisiología, Instituto de Investigaciones Biomédicas, Universidad Nacional Autónoma de México, México City 04510, Mexico; iraiz.nunez@iibiomedicas.unam.mx; 4 Hospital Central Militar, Dirección General de Sanidad SEDENA, Blvd. Manuel Ávila Camacho S/N, Lomas de Sotelo, México City 11200, Mexico; drgadu.sedena@gmail.com; 5 Servicio de Urología, Hospital Juárez de México, Instituto Politécnico Nacional 5160, Col. Magdalena de las Salinas, México City 07360, Mexico; omarhernandezleon@gmail.com

**Keywords:** miR-107, prostate cancer, castration-resistant

## Abstract

Prostate cancer (PCa) is a prevalent malignancy in men globally. Current diagnostic methods like PSA testing have limitations, leading to overdiagnosis and unnecessary treatment. Castration-resistant prostate cancer (CRPC) emerges in some patients receiving androgen deprivation therapy (ADT). This study explores the potential of circulating microRNA-107 (miR-107) in liquid biopsies as a prognosis tool to differentiate CRPC from non-castration-resistant PCa (NCRPC). We designed a case-control study to evaluate circulating miR-107 in serum as a potential prognosis biomarker. We analyzed miR-107 expression in liquid biopsies and found significantly higher levels (*p* < 0.005) in CRPC patients, compared to NCRPC. Notably, miR-107 expression was statistically higher in the advanced stage (clinical stage IV), compared to stages I–III. Furthermore, CRPC patients exhibited significantly higher miR-107 levels (*p* < 0.05), compared to NCRPC. These findings suggest that miR-107 holds promise as a non-invasive diagnostic biomarker for identifying potential CRPC patients.

## 1. Introduction

Prostate cancer (PCa) is the second most common malignancy in men globally, posing a significant health burden in Mexico [1,2]. In 2024, the Global Cancer Observatory (GLOBOCAN) estimated over 1.4 million new PCa diagnoses worldwide, with nearly 400,000 deaths, and Mexico reported over 26,000 new cases and over 7000 fatalities from PCa [1]. Late-stage diagnoses contribute to mortality rates, with approximately 60% of cases identified in advanced stages [3,4]. The prostate-specific antigen (PSA) test, although aiding early detection, can yield inconclusive results and has limited specificity, leading to over-diagnosis and unnecessary treatment of indolent PCa [3,4]. Additionally, some patients undergoing radical treatment for presumed localized PCa experience biochemical relapses, suggesting an underestimation of cancer aggressiveness at diagnosis [5]. The 5-year survival rate is nearly 100%, but once PCa cells spread into distant organs, such as bone, the overall survival rate of patients drops dramatically [6]. The median survival for metastatic PCa among patients younger than 70 years is 40 months [7]. This grim prognosis stems from factors such as the absence of initial symptoms, local invasiveness, and the potential for early-stage metastasis [5]. Androgen deprivation therapy (ADT), initially effective for many PCa patients, often leads to the development of metastatic castration-resistant prostate cancer (CRPC) within two years. This form of the disease presents a diagnostic challenge, despite the use of various proteomic and metabolomic biomarkers [8].

Recent discoveries highlight the potential of microRNAs (miRNAs) as diagnostic tools. These small, non-coding RNAs play crucial roles in cellular processes like proliferation, differentiation, and apoptosis, processes often disrupted in cancer [9,10,11,12,13,14]. Their unique expression profiles in cancer tissues, compared to normal tissues, make them promising biomarkers. Additionally, their stability in bodily fluids like serum (liquid biopsies) makes them suitable for minimally invasive testing [15,16,17,18,19]. The discovery of miRNAs unveiled their role in regulating fundamental cellular processes, and dysregulation in their expression has been linked to various human diseases, including cancer [16,17]. Advances have been made in understanding miRNA stabilization and detection in blood, establishing their presence in various bodily fluids [18,19]. Notably, circulating miRNAs are protected from degradation by binding to proteins or being encapsulated within vesicles [19,20,21,22]. These features make blood-based miRNAs, including those investigated in this study, attractive candidates for cancer detection, monitoring tumor dynamics, and assessing prognosis [23,24,25].

Several studies have reported associations between circulating miRNA levels and PCa [26,27]. One such miRNA, miR-107, has been shown to outperform PSA in differentiating between control and PCa patients [28]. However, its role in PCa progression after ADT remains unexplored. Other miRNAs, like miR-375, miR-378, and miR-141, are reported to be overexpressed in the liquid biopsies of CRPC patients [29]. This study aims to evaluate the expression levels of miR-107 in liquid biopsies from Mexican patients with confirmed ADT PCa, categorized by both cancer stage and castration resistance. We investigate the potential of circulating miR-107 levels as a potential biomarker for ADT PCa and CRPC

## 2. Results

### 2.1. Expression Profile of miR-107 in the Prostate Cancer Liquid Biopsies Dataset

**Figure 1:**
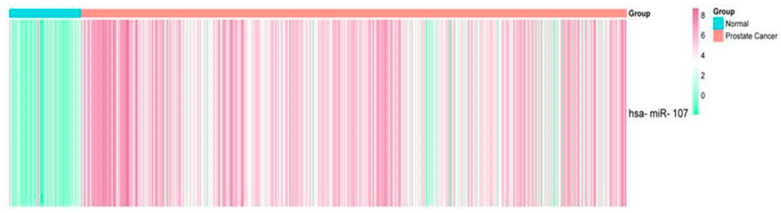
Expression profiles of miR-107 in liquid biopsies from patients with PCa vs. normal liquid biopsies dataset. A fold change of 1.5 and a *p*-value of < 0.05 were used as cut-off values.

To identify potential differentially expressed miRNAs (DEMs) in liquid biopsies from PCa patients, we analyzed public data from the GEO Omnibus dataset, GSE112264. Following the initial analysis, we filtered the identified DEMs to focus on those exhibiting similar expression patterns between PCa and normal (CTRL) liquid biopsies. A total of 809 PCa patients vs. 241 normal patients were found to consistently exhibit differential expression, with miR-107 overexpressed in the PCa group (fold change of 1.5, *p*-value < 0.05) (Figure 1). This allowed us to identify an overexpression of miR-107 in PCa liquid biopsies, suggesting that miR-107 could be used as a potential biomarker for PCa. These miRNAs have been previously implicated in PCa development and progression, and they could potentially be used to improve diagnosis or prognosis, which is addressed in the present study.

### 2.2. The miR-107 Expression Levels Are Higher in Liquid Biopsies from Cancer Patients with Androgen Deprivation Therapy

**Figure 2:**
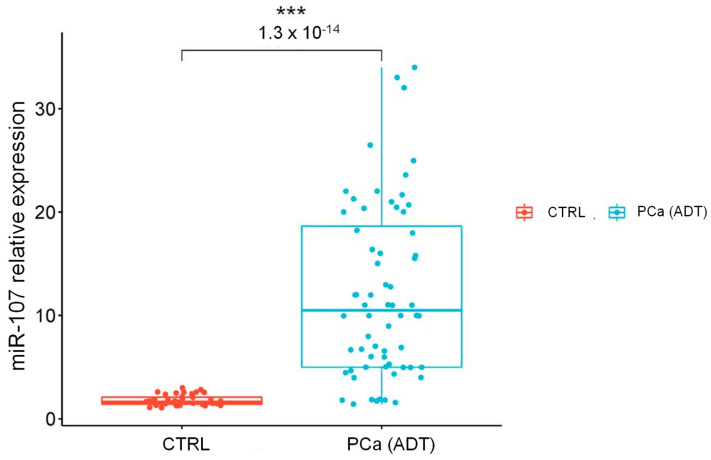
Relative expression of miR-107 in liquid biopsies from CTRL and ADT PCa patients. The relative levels of miR-107 that were determined by qRT-PCR and calculated using 2^−ΔΔCt^ are represented. The boxplot graph shows the median with quartile. Error bars represent the ±S.D. of three technical replicas. Mean comparison using a *t*-test. ***: *p* < 0.001. RNU6 was used for the normalization of the miR-107 levels.

To validate previous findings from publicly available data and to determine the clinical utility of miR-107, we analyzed liquid biopsies from Mexican patients with PCa treated with different ADTs (*n* = 63) and healthy controls (*n* = 42) who tested negative for PCa (CTRL). qRT-PCR assays were performed to analyze the expression profile of miR-107 in the liquid biopsies of PCa ADT patients and controls (Figure 2). These results revealed significant differences in miR-107 expression levels between the CTRL group and the PCa group, where the miR-107 expression level in the liquid biopsies of PCa patients had a 12-fold change, compared to the control group (*p* = 0.000012).

### 2.3. Stratification of Androgen Privation Resistance Prostate Cancer Cases in Mexican Patients

To investigate the potential correlation between miR-107 expression and the clinical–pathological features and aggressiveness of ADT PCa, the 63 patient cases were divided into diagnosed clinical stages (CSI, CSII, CSIII, and CSIV), and the descriptive information of the study groups is presented in Table 1.

**Figure 3:**
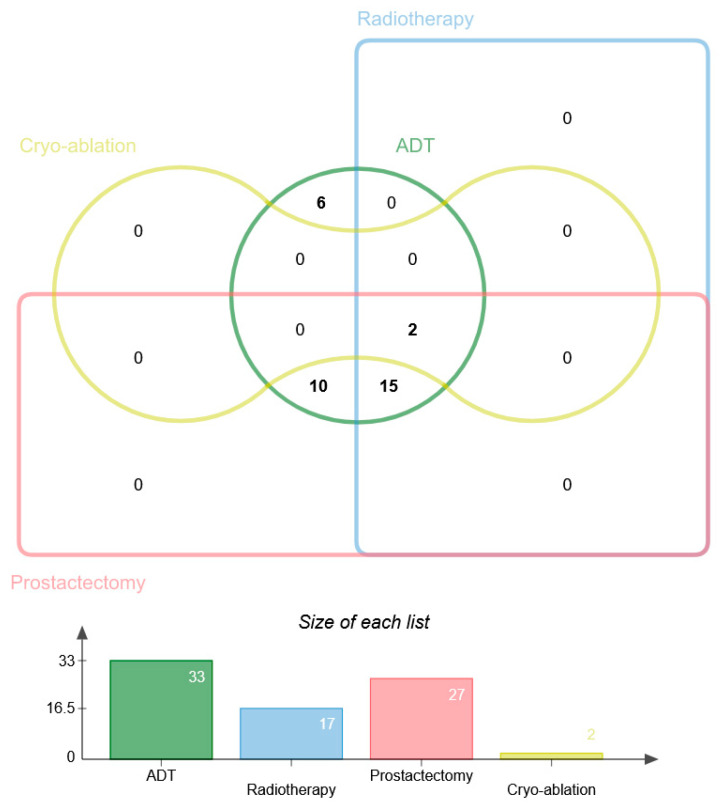
Correlation of additional treatment to ADT in PCa patients. The Venn diagram shows the additional treatments for ADT of PCa patients including radiotherapy, prostatectomy, and prostatic cryo-ablation. The numbers in the circles indicate the number of elements in the subsets based on presence/absence data. The numbers in the overlapping regions indicate the quantity of elements shared between subsets. The dataset indicates the size of each list analyzed.

All ADT PCa patients (*n* = 63) were treated with abiraterone acetate (*n* = 23), leuprolide acetate (*n* = 20), and goserelin (*n* = 20) distributed in all clinical stages and received complementary treatment as indicated: radiotherapy, radical prostatectomy, or cryotherapy. Only two patients were treated with surgery for symptom relief (prostate cryo-ablation). The main treatment used for PCa cases was ADT, combined with radiotherapy and surgery (prostatectomy and cryotherapy) (Figure 3).

### 2.4. Correlation of the Expression of miR-107 with the Clinical Stages of Androgen Privation Therapy Prostate Cancer and Liquid Biopsies

**Figure 4:**
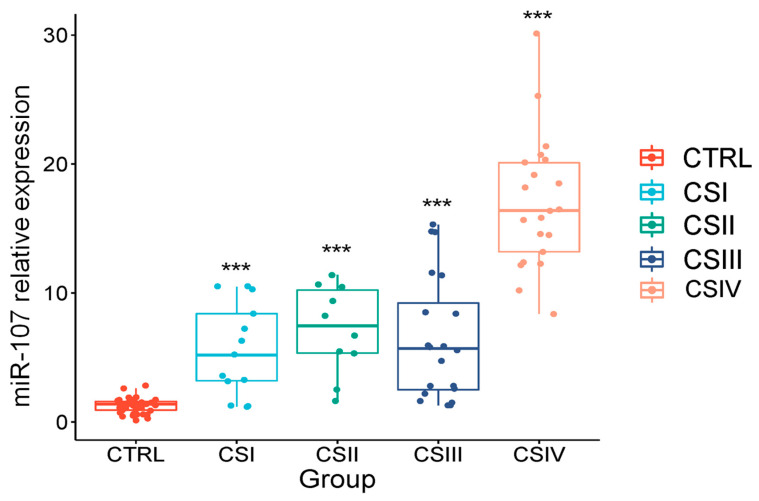
Relative expressions of miR-107 in liquid biopsies from ADT PCa patients and the clinical stages. The relative levels of miR-107 in liquid biopsies from patients with ADT PCa (*n* = 63) in different confirmed pathological clinical stages (CSI, CSII, CSIII, and CSIV) were measured by qRT-PCR and calculated using 2^−ΔΔCt^. The boxplot graph shows the median with the quartile. Error bars represent ±S.D. of the boxplot graph and displays the median with quartiles. Error bars represent ±S.D. from two independent experiments conducted in triplicate for each sample. Mean comparison was made using ANOVA and the Tukey test. ***: *p* < 0.001. RNU6 was used for the normalization of the miR-107 levels.

Once the miR-107 overexpression in liquid biopsies was determined, we investigated a potential association between miR-107 expressions in the clinical stages of ADT PCa cases at the moment of diagnosis (*n* = 63). Patient age at diagnosis ranged from 59 to 90 years (median 72), and body mass index had a median of 27.9. The ADT-treatment PCa cases (*n* = 63) were categorized by a clinical stage (CS) from one to four (CSI-CSIV) and healthy controls (*n* = 42). As shown in Figure 4, miR-107 expression in CSIV (*n* = 20) was significantly higher (*p* < 0.005), compared to CSI (*n* = 1 3, *p*-value 0.011), CSII (*n* = 10, *p*-value 0.032), and CSIII (*n* = 20, *p*-value 0.012). It is important to note that there was also a differential expression of miR-107 in all clinical stages of CRPC, compared to the control group (*p* < 0.001) (Figure 2). However, miR-107 exhibited a statistically significant pattern of differential expression in CSIV, compared to the other three clinical stages of ADT PCa, by mean comparison using ANOVA and the Tukey test. Nevertheless, further validation with larger cohorts is necessary to fully understand this expression pattern in ADT, as well as to elucidate the mechanisms by which miR-107 is regulated in the CSIV stage of PCa. However, further validation with larger cohorts is necessary to fully understand this expression pattern in ADT and is also needed to understand the mechanisms by which miR-107 is regulated in the CSIV PCa stage.

### 2.5. Circulating miR-107 as a Potential Biomarker for Castration-Resistant Prostate Cancer

We analyzed the specific miR-107 expression profiles in 33 CRPC patient samples and 30 NCRPC patient samples, previously shown to have higher overall miR-107 expression levels, compared to the CTRL group (Figure 1), to evaluate their potential as CRPC biomarkers. The distribution of clinical stages among CRPC patients is presented in Table 2.

**Figure 5:**
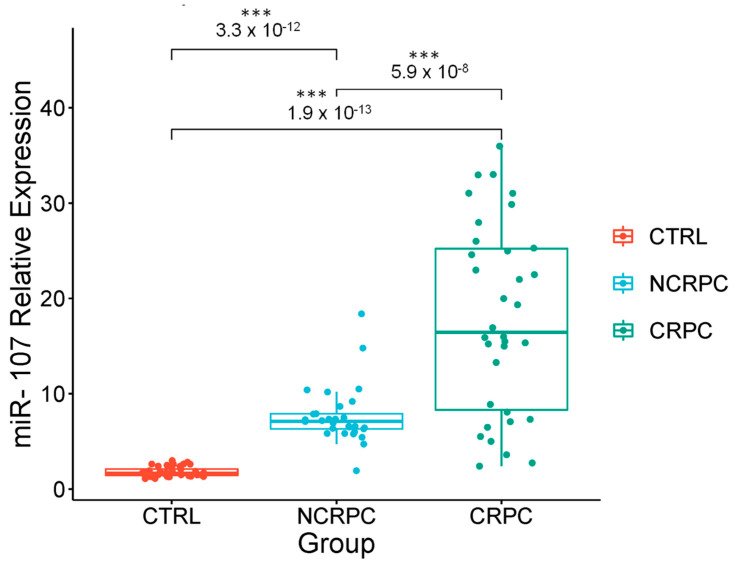
Relative expressions of miR-107 in liquid biopsies from CRPC in comparison with non-CRPC. The relative levels of miR-107 in liquid biopsies from patients with PCa CRPC and NCRPC by ADT were measured by qRT-PCR and calculated using 2^−ΔΔCt^. The boxplot graph displays the median with quartiles. Error bars represent ±S.D. from two independent experiments conducted in triplicate for each sample. Mean comparison was made using ANOVA and the Tukey test. ***: *p* < 0.001. RNU6 was used for the normalization of the miR-107 levels.

As depicted in Figure 5, miR-107 liquid biopsy expression level in CRPC patients was 14.1 ± 9.0, compared to 4.97 ± 2.19 in non-castration-resistant prostate cancer (NCRPC) (*p* = 5.9 × 10^−8^) patients and the control group (*p* < 0.001). This significantly higher miR-107 level in CRPC, compared with NCRPC patients and the CTRL group, suggests a possible role of miR-107 in the evolution of carcinoma biology. The high abundance of miR-107 in CRPC suggests its involvement in the pathogenetic mechanisms driving PCa progression toward castration-resistant disease. In this context, miR-107 could be considered a highly expressed miRNA in PCa, particularly in CRPC patients. Its potential application lies in CRPC late-stages detection, offering a novel miRNA biomarker to improve survival rates of PCa patients at risk of failing the standard ADT. This could allow for early intervention and the implementation of the most effective therapeutic methods for prevention.

**Figure 6:**
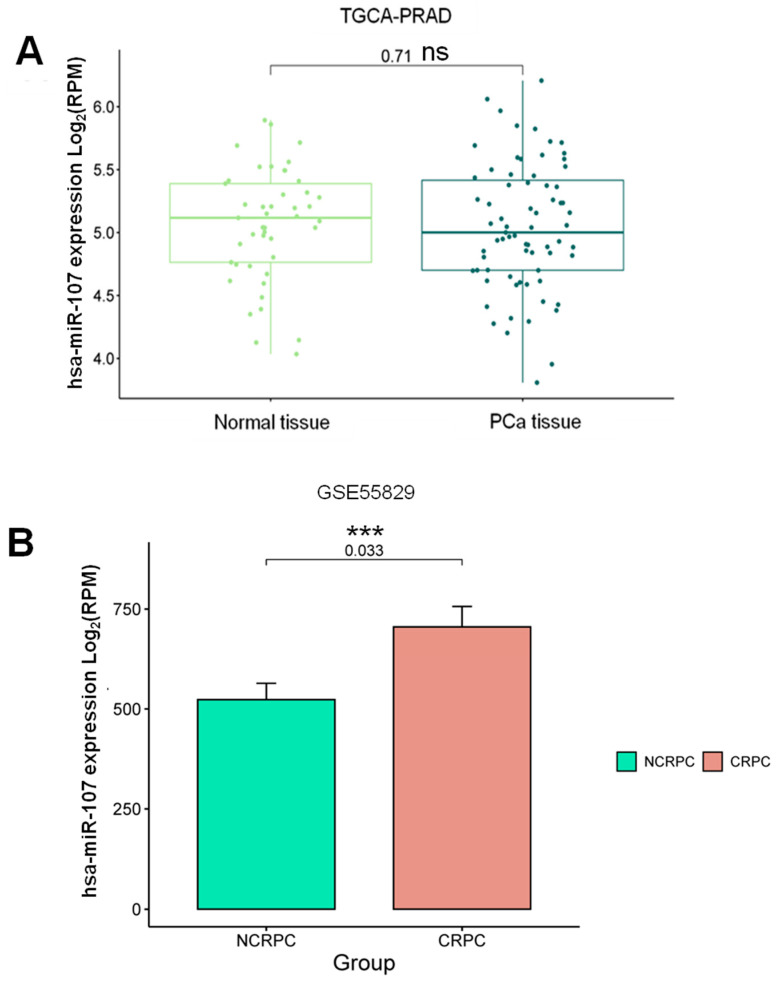
Expression profile of miR-107 in CRPC in comparison with non-CRPC tissue. (**A**) miR-107 profile expression obtained from the TCGA-PRAD database through the Xena Browser comparing normal tissue and PCa tissue. (**B**) miR-107 profile expression obtained from the GEO database, GSE55829, comparing xenograft NCRPC and CRPC tissue. Mean comparison of the log_2_ of reads per million (RPM) using the *t*-test. ***: *p* < 0.05. ns: not significant. A fold change of 1.5 and a *p*-value of <0.05 were used as cut-off values.

To investigate the potential correlation between circulating and tissue miR-107 in PCa patients, compared to benign prostatic hyperplasia (BPH), considered as normal tissues, and CRPC patients, we employed a multi-step approach. Initially, we utilized the TCGA Prostate Adenocarcinoma (TCGA-PRAD) database through the Xena Browser to analyze miR-107 expression in PCa and normal tissue samples. Interestingly, we observed no significant differences in miR-107 expression between BPH and PCa samples (*p* = 0.71) (Figure 6A). To further validate findings in androgen therapy, we retrieved public data on miR-107 expression in NCRPC and CRPC tissues (GSE55829). Remarkably, these data revealed a significant overexpression of miR-107 in CRPC tissue (*p* = 0.033) (Figure 6B). These findings support the notion that miR-107 could serve as a prognostic biomarker for CRPC patients, due to increased miR-107 levels in liquid biopsies, potentially originating from the primary tumor and influencing its development and progression to an advanced disease stage, and could be detected as a circulating marker for CRPC.

### 2.6. Evaluation of miR-107 as a Potential CRPC Diagnostic Biomarker

**Figure 7:**
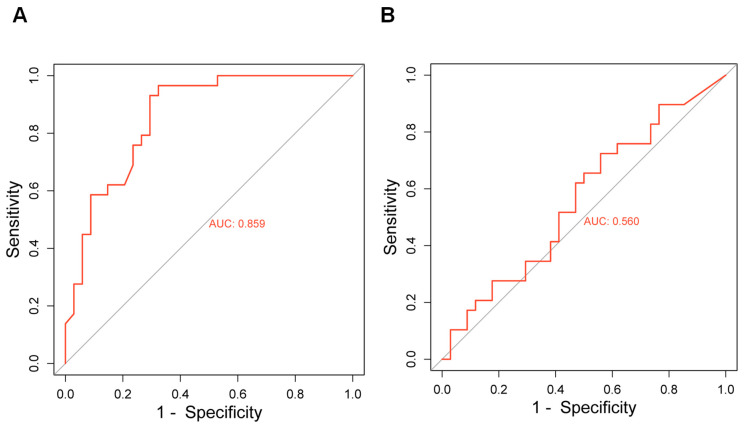
Evaluation of receiver operating characteristic (ROC) curves and area under the curve (AUC) in CRPC and NCRPC patients. (**A**) ROC and AUC values were calculated to assess the feasibility of using liquid biopsy miR-107 levels from CRPC and NCRPC patients as a potential diagnostic tool. (**B**) The AUC value was calculated for PSA.

To assess the diagnostic power of miR-107, we analyzed the expression of miR-107 to determine optimal cut-off points for differentiating advanced cancer patients with CRPC from those with NCRPC (Figure 7). We employed a receiver operating characteristic (ROC) curve, plotting sensitivity against the false positive rate (1-specificity) at various threshold levels of miR-107 and PSA. The area under the curve (AUC) for miR-107 was calculated as 0.85 (Figure 7A), indicating a good ability of miR-107 to discriminate between CRPC and NCRPC, compared with 0.56 for PSA (Figure 7B). At this threshold, the assay demonstrated a sensitivity of 89.0%, a specificity of 68.8%, and an overall accuracy of 89.0%. These findings suggest that miR-107 serum levels hold promise as a potential biomarker for distinguishing CRPC patients from NCRPC with high accuracy and significance. Compared to conventional prostate cancer markers, miR-107 offers clinically relevant detection capabilities. However, it is important to consider the positive predictive value (PPV) of this test in the context of overall disease prevalence. Further studies with larger patient populations are needed to determine the PPV and establish miR-107 as a reliable diagnostic tool for CRPC.

## 3. Discussion

Some of the currently used biomarkers for PCa remain highly invasive. For example, the prostate cancer antigen 3 (PCA3) is a biomarker only expressed in the prostate and can be detected in urine and prostatic fluid. While this does not require blood collection, it is considered more invasive than blood-based tests, as it requires a digital massage of the prostate before urine collection [9]. Due to this, the search for new biomarkers obtained through less invasive methods with faster and more specific results has recently generated interest. Most studied miRNAs play an important role in tumor development and cancer progression and, as shown here, are attractive for PCa detection and treatment. In the present study, we focused on the potential biomarker role of circulating miR-107 to evaluate its efficacy and accuracy in the detection of PCa progression stages, mainly in CRPC. The miR-107 has been linked to multiple cellular processes and pathways, especially those that regulate cell survival, such as autophagy, necrosis, ER stress, and cancer progression [30,31,32,33]. In this work, we reported that miR-107 is significantly overexpressed (*p* < 0.05) in ADT PCa patient liquid biopsies, compared to healthy control volunteers. Other studies have investigated the expression of miRNAs as potential biomarkers, such as Herrero-Aguayo et al. (2022), who reported 12 miRNAs differentially expressed in PCa patient plasma, compared to controls, and correlated with metastases. However, whether miR-107 could be used as a marker of PCa progression or treatment was not investigated [28]. In the context of the use of this miRNA as a potential biomarker, an analysis of urine samples indicated that miR-107 and miR-574-3p were significantly associated with PCa risk [26].

The PCa management has relied on the fundamental trio of PSA, histological Gleason score (GS), and clinical stage. However, this triad presents challenges in terms of sensitivity and specificity; discovering less invasive biomarkers specific to progression is essential to avoid overdiagnosis and overtreatment. In our study, statistically significant differences (*p* < 0.05) were found between ADT PCa clinical stages CSI, CSII, and CSIII and clinical stage CSIV, which showed higher levels ofmiR-107 in liquid biopsies. The miR-107 family has been linked to tumor progression from initial to advanced PCa stages, and our results are consistent with these reports [34,35,36,37]. miR-107 was significantly overexpressed (*p* < 0.001) in CRPC liquid biopsies, compared to NCRPC, in both ADT patients. In the CRPC context, other studies have proposed prognostic values of both miR-141 and miR-375, with a trend of increasing plasma levels at disease progression from low-risk PCa, high-risk PCa, and metastatic CRPC [38]. To our knowledge, this is the first report of miR-107 association with CRPC, which is the end stage of a multifactorial and heterogeneous disease process [39,40,41]. Significant progress has been made in characterizing the molecular basis of CRPC in recent years. However, despite these advances, resistance to PCa therapies remains poorly understood [42,43]. The heterogeneity of CRPC is a well-established clinical challenge; therefore, basing patient stratification on metastatic tissue biopsies is currently not a valid option, since the methods are invasive [44].

To gain accurate and novel insight into the role of miR-107 in PCa and its possible use as a biomarker for CRPC, we also evaluated the ROC curve and determined the AUC value. Our data indicate an AUC of 0.85, with an optimal relative expression cut-off point of about 1.2, a high sensitivity of 89.0%, and a specificity of 68.8%. Our study provides 89.0% accuracy in measuring miR-107 serum levels to detect CRPC. This high accuracy demonstrates that this method is a potentially viable option for the early diagnosis of CRPC. The measurement of miR-107 can also be used to identify additional prognostic factors in the initial stage of CRPC development. In this context, miR-107 levels could potentially be determined at the time of diagnosis to identify patients with aggressive disease/micrometastases and/or to predict recurrence following primary ADT. However, one of the limitations of this study is the relatively small sample size; although the results are consistent, confirmation with larger cohorts would be necessary before considering translational application in a clinical setting to improve stage diagnostic accuracy and to potentially guide personalized treatment strategies for PCa patients.

The AUC values of miR-107 obtained in our study compared to the PSA of PCa patients after ADT reflect data from our specific sample and could vary in studies with larger cohorts. While studies such as the one by Catalona et al. (1994), which involved a larger cohort, reported an AUC of 0.72 for PSA in non-treated PCa patients [45], our cohort is significantly smaller but specific to ADT samples. Generally, an extremely high PSA level can potentially indicate metastatic prostatic adenocarcinoma, but it is not a definitive sign [46]. Understanding these variations is crucial for evaluating miR-107’s potential as a treatment-resistance biomarker.

Additionally, it is necessary to understand the molecular mechanisms by which miR-107 could contribute to the development and progression of CRPC. Recently, Liang et al. reported that miR-107 induces chemoresistance in colorectal cancer (CRC) through the CAB39–AMPK–mTOR pathway, promoting metastasis [47]. Contrary to our findings, Mihelich et al. found that miR-107 was downregulated in high-grade PCa serum. This divergence underscores the complexity of miR-107’s role in PCa, ADT, and CRPC, necessitating further research to clarify its function. These discrepancies in expression profiles could be due to differences in specific molecular contexts, technical variations in detection and normalization methods, patient populations, experimental conditions, or stages of PCa [48]. At least in vitro, miR-107 overexpression altered aggressiveness in PCa cells, inhibiting proliferation by the G1/S phase arrest targeting cyclin E1, migration, and tumorsphere formation, but did not affect apoptosis or cell motility, modulating the expressions of genes involved in disease pathophysiology and acting as a tumor suppressor [28,49]. The carcinogenic or cancer-suppressor effects of miR-107 and its functions can be used as potential diagnostic and prognostic biomarkers or targets for therapeutic intervention, even in conjunction with other pathologies, such as obesity [28]. In our TCGA analyses, we found no significant differences in miR-107 expression levels between normal and PCa tissues, but miR-107 was reported to be downregulated in PCa tissue, compared with normal prostate cells and peritumoral tissues [49].

The discrepancy between TCGA-PRAD data and our results in liquid biopsies could be attributed to treatment status, as treatment may influence miR-107 levels in tissue samples; also, there are reports in metastatic PCa cohorts where there was a significant negative correlation of miRNAs between tumor tissue expression and plasma levels [50]. These findings support the notion that miR-107 could serve as a prognostic biomarker for CRPC patients. Elevated miR-107 levels, potentially originating from the primary tumor and influencing its development and progression to a late stage, could be detected as a circulating marker for treated PCa. Previous studies have demonstrated the prognostic value of other miRNAs, among which are miR-141 and miR-221, which have been associated with lower overall survival (OS) in patients treated with abiraterone [38].

Taken together, this study provides preliminary evidence supporting the potential of miR-107 as a biomarker for CRPC after ADT. Broader investigations are needed to validate these findings and to elucidate the role of miR-107 in the biology of PCa and treatment resistance.

## 4. Materials and Methods

### 4.1. Data Acquisition of PCa and CRPC from Liquid Biopsies and Tissue

Microarray data for PCa and normal liquid biopsy samples were obtained from the GEO Omnibus database. The following dataset was used: GSE112264 for miRNA expression. Limma package in the R statistical environment was used to identify differentially expressed miRNAs (DEMs) within each dataset.

To evaluate miR-107 expression in prostate PCa tissue samples, we utilized the TCGA Prostate Adenocarcinoma (TCGA-PRAD) database through the Xena Browser (accessed on 1 July 2024). This allowed us to compare miR-107 expression levels between PCa and normal tissue samples to identify any significant differences. Additionally, we retrieved public data on miR-107 expression in benign prostatic hyperplasia (BPH) tissues (GSE14392), treatment-naïve PCa tissues (GSE59156), and castration-resistant PCa xenograft tissues (GSE55829). We then analyzed miR-107 expression levels in each of these groups and compared them to the TCGA-PRAD data. Limma package in the R statistical environment was used to identify DEMs within each dataset. A fold-change threshold of 1.5 (absolute value) and a *p*-value cut-off of less than 0.05 were used to define statistically significant differences in miRNA expression.

### 4.2. Liquid Biopsies from NCRPC, CRPC Patients, and Controls

Liquid biopsies from NCRPC patients (*n* = 30), CRPC patients (*n* = 33) (total PCa ADT *n* = 63), and the healthy control (*n* = 42) were obtained from recruited volunteers who met the inclusion criteria at Hospital Central Militar of Mexico City and Hospital Juárez de México. The study was approved by the ethics and research committees (AEEI-79020 and HJM 009/23-I, respectively). After signed informed consent, 4–6 mL blood samples (liquid biopsies) were collected in BD Vacutainer^®^ serum tubes (Beckton-Dickinson, Franklin Lakes, NJ, USA), centrifuged at room temperature (300× *g* for 5 min) to separate the serum, aliquoted, and frozen at −80 °C within 24 h of sampling. Inclusion criteria for cases were: males over 40 years old with a confirmed diagnostis of PCa with ADT, PSA > 4 ng/mL^−1^, and Gleason score ≥ 6. Exclusion criteria for cases were: male patients under 40 years old, males with a family history of PCa, males with a PSA diagnosis < 4 ng/mL, and patients with a previous diagnosis of another type of cancer or catastrophic diseases. Controls were healthy males over 40 years old, with PSA < 4 ng/mL^−1^ and no family history of PCa or catastrophic diseases. Hemolyzed serum samples were excluded from both groups (cases and controls). The samples were frozen at −80 °C until use.

### 4.3. RNA Isolation from Liquid Biopsies

Total RNA for miR-107 expression analysis was extracted from 200 µL of liquid biopsies of the analyzed groups. Trizol (1 mL) was added and mixed thoroughly by pipetting, and then the lysate was transferred to a 1.5 mL microcentrifuge tube. The mixture was incubated at room temperature for 5 min, followed by the addition of 200 µL of chloroform per mL of Trizol reagent, and shaken vigorously for 15 s. After a 3 min incubation at room temperature, the tube was centrifuged at 12,500 rpm for 25 min at 4 °C. The aqueous phase was carefully collected into a fresh 1.5 mL microcentrifuge tube. RNA precipitation was then carried out by adding 500 µL of isopropanol, with the pellet incubated on ice for 20 min. The sample was centrifuged again at 12,500 rpm for 25 min at 4 °C. After removing the supernatant, the pellet was washed with 1 mL of 75% ethanol and allowed to air dry for 5 min. Finally, the RNA pellet was resuspended in 20 µL of nuclease-free water. The RNA concentration was measured using spectrophotometry, and the RNA was subsequently used for cDNA synthesis.

### 4.4. cDNA Synthesis and qRT-PCR

Complementary DNA (cDNA) was obtained from isolated RNA from liquid biopsies using the MicroRNA Reverse Transcription Kit (Applied Biosystems, Foster City, CA, USA). All reactions were performed according to the manufacturer’s recommendations. cDNA samples were kept at −80 °C until the application of quantitative real-time polymerase chain reaction (qRT-PCR) with specific primers for miR-107 (forward primer 5′-GCTTCTTTACAGTGTTGCC-3′ and reverse primer 5′-TCTGTGCTTTGATAGCCCTGT-3′) and U6 snRNA (RNU6) (forward primer 5′-CTCGCTTCGGCAGCACA.3′ and reverse primer 5′-AACGCTTCACGAATTTGCGT-3′) using SYBR Green assay (Applied Biosystems), according to the supplier instruction for using the Applied Biosystems 7500 Real-Time PCR system. All the assays were performed in duplicate with three technical replicas.

### 4.5. Statistical Analysis

To identify DEMs within each dataset, the limma package in the R statistical environment was employed. A fold-change threshold of 1.5 (absolute value) and a *p*-value cut-off of less than 0.05 were used to define statistically significant differences in miR-107 expression. Differences in miRNA expression between the case and control groups were assessed using an ANOVA, a post hoc test (Tukey), and t-test, with *p* < 0.001 being considered statistically significant. Differences in miR-107 expression were estimated using the 2^−ΔΔCt^ equation and expressed as fold changes, normalized to snRNU6 (endogenous control) in liquid biopsies [51,52,53]. The diagnostic power of miR-107 expression was evaluated based on sensitivity, specificity, and cut-off values obtained from receiver operating characteristic (ROC) curves. The scores showing statistical significance are indicated in the figures with asterisks. The corresponding values are indicated in the figure legends. Statistical analyses were performed using GraphPad software (v9.3.0).

## 5. Conclusions

Our findings support the growing evidence suggesting that non-coding RNAs, such as miRNAs, hold promise as non-invasive biomarkers for PCa diagnosis. This study demonstrates that circulating miR-107 has a higher expression in CRPC patients’ liquid biopsies, compared to NCRPC and healthy controls. Furthermore, our data suggest that miR-107 may be differentially expressed in the progression of primary tumors to metastatic disease in PCa with ADT. These results are consistent with the established role of miR-107 in cancer-associated signaling pathways and its potential contribution to aggressive CRPC development. Importantly, our research indicates that miR-107 measurement has the potential to accurately detect androgen-resistant prostate cancer for treatment. This non-invasive approach could significantly improve diagnostic strategies and contribute to the growing clinical utility of miRNAs in PCa treatment management.

## Figures and Tables

**Table 1:** Clinical stage Gleason scores (GS) of ADT PCa cases.

Clinical Stage	CS Cases (*n*)	Cases (*n*)	Gleason Score ^1^
CSI	13	6	6 (3 + 3)
		4	7 (4 + 3)
		3	8 (4 + 4)
CSII	10	1	6 (4 + 2)
		6	7 (4 + 3)
		1	8 (4 + 4)
		2	9 (5 + 4)
CSIII	20	12	7 (4 + 3)
		5	6 (4 + 2)
		3	8 (4 + 4)
CSIV	20	8	9
		2	8
		8	10
		1	7
		1	6

^1^ Results are reported as the GS, which indicates the aggressiveness of the cancer detected by the pathologist, according to the AJCC Cancer Staging Manual (https://www.facs.org/media/j30havyf/ajcc_7thed_cancer_staging_manual.pdf (accessed on 5 July 2024).

**Table 2:** Clinical stages and Gleason scores of CRPC patients used in this study.

Clinical Stage	Number of Patients (*n*)	Gleason Score (*n*)
CSI	10	6 (6), 7 (4)
CSII	2	9 (2)
CSIII	3	8 (3)
CSIV	18.	9 (8), 10 (8), 7 (2)
Total	33	33

CS: Clinical stage.

## Data Availability

The original data presented in the study are openly available at [https://portal.gdc.cancer.gov/projects/TCGA-PRAD], and GEO omnibus datasets GSE112264 and GSE55829 are available at [https://www.ncbi.nlm.nih.gov/geo/], accessed on 5 July 2024.
